# Comparative metabolic study of the chloroform fraction of three *Cystoseira* species based on UPLC/ESI/MS analysis and biological activities

**DOI:** 10.1080/14756366.2023.2292482

**Published:** 2023-12-12

**Authors:** Shaza H. Aly, Ahmed M. Elissawy, Mahmoud A. El Hassab, Taghreed A. Majrashi, Fatma E. Hassan, Eslam B. Elkaeed, Wagdy M. Eldehna, Abdel Nasser B. Singab

**Affiliations:** a Department of Pharmacognosy, Badr University in Cairo (BUC), Badr City, Egypt; b Department of Pharmacognosy, Ain-Shams University, Cairo, Egypt; c Centre of Drug Discovery Research and Development, Ain Shams University, Cairo, Egypt; d Department of Medicinal Chemistry, King Salman International University (KSIU), South Sinai, Egypt; e Department of Pharmacognosy, College of Pharmacy, King Khalid University, Asir, Saudi Arabia; f Department of Physiology, General Medicine Practice Program, Batterjee Medical College, Jeddah, Saudi Arabia; g Medical Physiology Department, Kasr Alainy, Cairo University, Giza, Egypt; h Department of Pharmaceutical Sciences, College of Pharmacy, AlMaarefa University, Riyadh, Saudi Arabia; i Department of Pharmaceutical Chemistry, Kafrelsheikh University, Kafrelsheikh, Egypt

**Keywords:** Antioxidants, anti-inflammatory, antihyperglycemic, brown algae, molecular docking

## Abstract

This study aims to investigate the phytoconstituents of the chloroform fraction of three *Cystoseira* spp. namely *C. myrica*, *C. trinodis*, and *C. tamariscifolia* using UPLC/ESI/MS technique. The results revealed the identification of 19, 20 and 11 metabolites in *C. myrica*, *C. trinodis*, and *C. tamariscifolia,* respectively mainly terpenoids, flavonoids, phenolic acids and fatty acids. Also, an *in vitro* antioxidant study using FRAP and DPPH assays was conducted where the chloroform fraction of *C. trinodis* displayed the highest antioxidant activity in both assays, which would be attributed to its highest total phenolics and total flavonoids. Besides, the investigation of COX-1, *α*-glucosidase and *α*-amylase inhibitory activities were performed. Regarding *C. trinodis,* it showed the strongest inhibitory activity towards COX-1. Moreover, it showed potent inhibitory activity towards *α*-glucosidase and *α*-amylase enzymes. According to the molecular docking studies, the major compounds characterised showed efficient binding to the active sites of the target enzymes.

## Graphical Abstract

**Figure:**
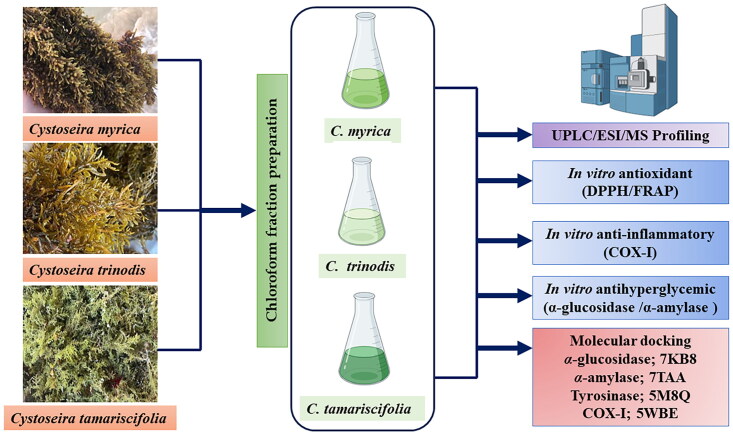

## Introduction

In recent times, marine organisms have emerged as a crucial source of bioactive compounds that possess remarkable biological effects owing to their diverse nature. One particular class of marine organisms, namely macroalgae or seaweeds, has gained significant importance in this regard due to its abundance of bioactive compounds. The broad range of biological activities exhibited by marine macroalgae makes it one of the most significant groups in the marine ecosystem^1–4^. Marine macroalgae exhibited a plethora of biological effects such as antioxidant, anti-inflammatory, cytotoxic, antimicrobial and antidiabetic effects^2^^,^^5–7^. In addition, macroalgae produce different secondary metabolites of mixed biogenesis with a vast range of structural diversity to protect themselves and as a defense mechanism against their environment such as terpenoids and phenolics^1^^,^^8^.

Genus *Cystoseira* is one of the famous macroalgae that belongs to Phaeophyta (brown macroalgae), family Sargassaceae and the order Fucales that comprises about 50 species worldwide that are distributed across subtropical waters, with the largest diversity and abundance of species found in the Mediterranean Sea^9^^,^^10^. Previous studies on members of the *Cystoseira* genus have led to the discovery of a diverse range of biological activities, including antioxidant, anti-inflammatory, cytotoxic, antidiabetic, and antimicrobial properties^1^^,^^5^^,^^6^^,^^11^. Besides, *Cystoseira* brown macroalgae possess different secondary metabolites specifically terpenoids, diterpenes, meroterpenoids and flavonoids^12–16^. Recently, the dependence on natural products to manage several diseases is of great importance. For example, natural secondary metabolites have the potential to regulate blood sugar levels and serve as effective antihyperglycemic agents through inhibition of key enzymes α-glucosidase and α-amylase^17^^,^^18^. Besides, the quest for alternative anti-inflammatory drugs and medications has led to a focused search among marine seaweed. Natural marine products that exhibit both anti-inflammatory and antioxidant activities have garnered significant attention due to their potential for pharmacological use^19–21^.

The *Cystoseira* thallus consists of enduring base components, namely the holdfast and the main axis, which are predominantly flattened into 'foliar expansions’ or basal leaves. These leaves can either be spiny or leaf-like. The receptacles, found at the tips of branches above the aerocysts, are the fertile sections of the annual thallus that may contain air vesicles. The number of secondary axes increases exponentially with main axis length, suggesting environmental constraints may limit central axis growth within a 44 cm size at the greatest frequency. These algae possess not only chlorophyll pigments, but also xanthophyll and fucoxanthin pigments. The brown colour of brown seaweeds is attributed to the presence of xanthophyll and fucoxanthin pigments^22–25^. The colour of *Cystoseira* varies across different species and undergoes some degree of variation over the lifespan of the thallus. Certain species, like *C. myrica*, exhibit a dark brown colour. Species like *C. trinodis* and *C. tamariscifolia* also have a greenish-blue brown colouration^22^^,^^26^. The photos of the three *Cystoseira* spp. Under study are illustrated in the (Figure S1).

Therefore, we continue our ongoing exploration for secondary compounds derived from *Cystoseira* spp. In this study, we present the results of our investigation of the chloroform fraction phytochemical composition and variations between three *Cystoseira* spp.; namely *C. myrica*, *C. trinodis*, and *C. tamariscifolia* along with their antioxidant activity, inhibitory effects against *α*-glucosidase, *α*-amylase and cyclooxygenase I enzymes. Furthermore, the study intends to assess the binding affinities between the identified major components and the tested enzymes using molecular docking studies.

## Materials and methods

### Plant material

*Cystoseira myrica*, *C. trinodis* and *C. tamariscifolia*, three samples of brown algae were collected from the shores of the Gulf of Suez in Ras Sedr city, Egypt 29°50′19.7′N and 32°37′36.8′E in October 2020. A voucher sample was identified according to Ibraheem et al.^27^ and deposited at the Pharmacognosy Department, Faculty of Pharmacy, Badr University in Cairo under code of BUC-PHG-CM-3, BUC-PHG-CT-4 and BUC-PHG-CT-5, respectively. To prevent evaporation, the samples were transported to the laboratory using sterile plastic bags filled with seawater. Subsequently, the algae were carefully rid of epiphytes and rock debris, and any surface salts were removed through a brief rinsing with freshwater.

### Preparation of the plant extract

The grounded air-dried *C. myrica*, *C. trinodis* and *C. tamariscifolia*, (100 g) were macerated in methanol three times at room temperature followed by concentration by evaporation under reduced pressure to yield a dark brown residue. Separately, the crude extract of each species was suspended in distilled water and fractionated using chloroform to yield the chloroform fraction that was concentrated under reduced pressure an low temperature (40 °C) using a rotary evaporator (Hei-VAP Value, Heidolph) to yield a dark brown sticky residue that weight for each sample 1.15, 1.35 and 1.30 g, respectively that was stored at 4 °C for further analysis^28–30^.

### UPLC-ESI-MS analysis

Metabolite profiling of the three species chloroform fraction was carried out using UPLC-ESI-MS analysis at the Centre of Drug Discovery Research and Development, Department of Pharmacognosy, Faculty of Pharmacy, Ain Shams University, Egypt, according to the previously reported method^3^^,^^31^. The experimental procedure involved utilising a Waters®TQD UPLC-MS instrument equipped with an ESI source and a Waters® acquity UPLC RP-C18 column, (100 × 2 mm, ID), and featuring a particle size of 1.7 µm. Additionally, an integrated precolumn was employed during the analysis. A gradient of acetonitrile and water was applied, ranging from 2% to 100%, in addition to 0.1% formic acid. One run took 35 min, with a flow rate of either 1 or 0.5 ml/min. For ESI-, the MS was run at −10 V, with a source temperature of 240 °C. High purity N_2_ was used as an auxiliary gas and sheath at 80 and 40 (arbitrary units) flow rates, respectively. The volume of the injection was 5 *µ*L. The spray voltage utilised was 4.48 kilovolts (kV), the tube lens voltage was 10.00 volts (V), and the capillary voltage was 39.6 V. The samples were dissolved in methanol of HPLC grade and subsequently filtered using a PTFE (polytetrafluoroethylene) membrane with a pore size of 0.2 *µ*m. The mass spectrometry analysis was conducted within the *m/z* range of 100 to 1200. Tentative identification of metabolites was attained by comparing the mass and spectral data of the identified compounds, obtained under negative ionisation mode, with previously reported data from the genus and the family and online public databases. The acquisition and analysis of data were performed using the XcaliburTM 2.0.7 software (Thermo Scientific, Karlsruhe, Germany).

### Total phenolics and flavonoids content

The quantification of both total phenolic (TPC) and flavonoid contents (TFC) in the chloroform fractions was carried out using spectrophotometric techniques, specifically the Folin-Ciocalteu^32^^,^^33^ and AlCl_3_ methods^34^, respectively. Briefly, to determine the TPC, the Folin-Ciocalteau method was employed, involving the mixing of 5 *µ*L of plant extract with a volume of 3 ml of Folin-Ciocalteau reagent (10%) and 0.8 ml of sodium bicarbonate (7.5%).

Following the mixing of the reaction solution, it was incubated at room temperature for 30 min. The absorbance of the resulting mixture was then measured at 765 nm using a Milton Roy (Spectronic 1201) spectrophotometer. The TPC was expressed as mg gallic acid equivalents (GAE)/g extract. For the TFC analysis, 0.1 ml of the extract was mixed with 3.90 ml of distilled water, followed by the addition of 0.3 ml of a 5% sodium nitrite solution. After allowing the mixture to react for 5 min, 0.3 ml of a 10% aluminium chloride solution was added. The resulting mixture was allowed to react for an additional 6 min. Subsequently, 2 ml of 1 mM^−1^ sodium hydroxide solution was added to the mixture, followed by the addition of 2.4 ml of distilled water to all samples. The absorbance of the samples was measured at 510 nm using a Milton Roy (Spectronic 1201) spectrophotometer, with a sample blank without any reaction serving as the reference. The TFC of the extracts are expressed as mg quercetin equivalents (QE)/g extract.

### Biological evaluation

#### In vitro assessment of antioxidant activity using DPPH and FRAP assays

Two methods were employed in this study to evaluate the antioxidant potential of the chloroform fractions of the three species under study. These included the use of 1,1-diphenyl-2-picrylhydrazyl (DPPH) radical scavenging assay^35^^,^^36^ and the ferric ion reducing antioxidant power (FRAP) assay^37^.

##### DPPH radical scavenging assay

In DPPH Radical Scavenging Activity, A freshly prepared methanol solution of 2,2-diphenyl-1-picrylhydrazyl (DPPH) radical with a concentration of 0.004% w/v was prepared and kept in the dark at a temperature of 10 °C. The tested fraction was dissolved in methanol to prepare a sample solution. Followed by, a 40 *µ*L volume of this sample was added to 3 ml of the DPPH solution. The absorbance readings were taken immediately using a UV-visible spectrophotometer (Milton Roy, Spectronic 1201). The decrease in absorbance at 515 nm was monitored continuously, with data being recorded at 1-min intervals until the absorbance reached a stable level. The absorbance of the DPPH radical solution without the presence of any antioxidant (control) and the reference compound ascorbic acid were also investigated. All measurements were conducted in triplicate, and the obtained values were averaged. The percentage inhibition (PI) of the DPPH radical was calculated according to the formula:

$$\mathrm{PI}=100\text{ X }[\frac{AC-AT}{AC}]$$

Where *A*C = Absorbance of the control at t = 0 min and *A*T = absorbance of the sample + DPPH at t = 16 min. The 50% inhibitory concentration (IC_50_), the concentration required to inhibit DPPH radical by 50%, was estimated from graphic plots of the dose response curve.

##### FRAP scavenging assay

In Ferric reducing antioxidant power (FRAP), the conversion of ferric ions to ferrous ions by the extract indicates its potential antioxidant activity. This method involves the use of different concentrations of the extract sample to reduce ferricyanide. Samples in 1 ml of methanol were mixed with 2.5 ml of 0.2 M sodium phosphate buffer (pH 6.6) and 2.5 ml of potassium ferricyanide [K_3_Fe (CN)_6_] (1%, w/v). Following a 20 min incubation at 50 °C, the reaction mixture was treated with 2.5 ml of trichloroacetic acid (10%, w/v). The reaction mixture was centrifuged, and the supernatant solution (2.5 ml) was mixed with 2.5 ml of deionised water and 0.5 ml of freshly prepared ferric chloride (0.1%, w/v). The resulting solution’s absorbance was measured at 700 nm, with a blank serving as the reference, using a spectrophotometer (Milton Roy, Spectronic 1201). Ascorbic acid was used as reference standard. The reducing capability percentage (%) was calculated as follows, according to Canabady-Rochelle et al. 2015^38^.

$$\text{Reducing capability }(\%)=100-[\frac{A0-As}{A0}\;x\;100]$$

A0: absorbance of the control solution and As: sample absorbance.

#### In vitro assessment of anti-hyperglycaemic activity

##### *α*-amylase inhibition assay

The method used to evaluate the *a*-amylase inhibitory activity of the tested chloroform fractions was based on a previously reported procedure and involved the use of BioVision’s *α*-amylase inhibitor screening kit (K482-100)^39–41^. In this experiment, 500 µL of both test samples and acarbose (1000–7.81 μg/mL) were combined with a 0.20 mM phosphate buffer solution (pH 6.9) containing a 0.5 mg/mL concentration of α-amylase. After mixing, the resulting solution was incubated at 25 °C for 10 min. Subsequently, 500 µL of a 1% starch solution in 0.02 M sodium phosphate buffer (pH 6.9) was introduced into each tube. The reaction mixtures were left to incubate at 25 °C for 10 min, after which the reaction was stopped by adding 1.0 ml of the 3,5-dinitro-salicylic acid colour reagent. Afterward, the test tubes were incubated in a boiling water bath for 5 min, then allowed to cool to room temperature. Following this, the reaction mixture was diluted by adding 10 ml of distilled water, and the absorbance was measured at 540 nm. For the control group, water was used in place of the extracts to represent 100% enzyme activity. All measurements were conducted in triplicate, and the resulting values were expressed as the mean ± standard deviation (SD). Using Acarbose as a standard drug and the concentration of the α-amylase and *α*-glucosidase inhibitor required to inhibit 50% of its activity under the conditions of the assay was determined as the IC_50_ value.

##### *α*-glucosidase inhibition assay

The method used to evaluate the *a*-glucosidase inhibitory activity was based on a previously reported procedure and involved the use of BioVision’s *α*-glucosidase inhibitor screening kit (K938-100)^42^. To begin the experiment, approximately 10 *µ*L of the extract being tested was combined with glutathione (10 *µ*L), α-glucosidase solution (10 *µ*L) in a phosphate buffer solution (pH 6.8), and PNPG (4-nitrophenyl-α-D-glucopyranoside) (10 *µ*L) in a 96-well microplate. The resulting mixture was then incubated for 15–20 min at room temperature.

In a similar manner, a blank was prepared by adding the sample solution to all the reaction reagents except for the α-glucosidase solution. The reaction relied on the activity of an active α-glucosidase enzyme to break down a synthetic substrate (PNPG), which results in the release of a chromophore (*p*-nitrophenol; OD = 410 nm). The reaction was halted by adding 50 *µ*L of 0.2 M sodium carbonate. The absorbance of both the tested oil and the blank was measured at 410 nm. The absorbance value of the blank was subtracted from that of the tested oil, and the resulting value was used to determine the IC_50_ value.

#### Assessment of anti-inflammatory activity (cyclooxygenase inhibition assay)

The cyclooxygenase inhibition assay was conducted using COX-1 Inhibitor Screening Kit (Catalog number K548-100, Milpitas, CA 95035 USA). The assay was carried using different concentrations of the tested sample (from 0.5 to 1000 *μ*g/mL, in a geometric sequence) were preincubated with the cyclooxygenase enzyme (COX-1) at room temperature (25 °C) for 5 min in the presence of haematin. Phenol (500 *μ*M), 1-leuco-dichlorofluorescein (20 *μ*M), arachidonic acid (50 *μ*M) and haematin (1 *μ*M) in 1 ml of 0.1 M Tris-buffer (pH 8) were pre-mixed and added to the enzyme mixture. The spectrophotometric measurement was performed using a Milton Roy (Spectronic 1201) instrument at 502 nm, and the absorbance was recorded within 15 s of the sample addition. A reference standard of Celecoxib was used, and a control sample was prepared without the addition of enzymes. The IC_50_ value (μg/mL), which is the concentration of the extract that inhibited 50% of cyclooxygenase -I that was calculated from the curve^43^.

### 2.6. Molecular Modelling

In the present work, Vina, Autodock and MGL tools were implemented for conducting the molecular docking. Sixteen major compounds of the studied *Cystoseira* spp. were sketched and converted into 3D structures using Discovery Studio. The X-ray crystallographic structures of *α*-amylase, *α*-glucosidase, COX-1 & tyrosinase enzymes were downloaded from protein data bank (PDB) (www.pdb.org) accessed on 15 July 2023 using the following IDs: 7TAA, 7KB8, 5WBE and 5M8Q^44–47^, respectively. Docking investigations were performed using MOE 2019^48^^,^^49^, which was also employed to generate 2D interaction diagrams illustrating the docking of ligands with potential targets. Each protein file’s co-crystallized ligand was used to identify the binding site of the enzymes where the docking was conducted. Finally, 16 major compounds of the extract were docked using triangle matcher as a placement method and London dG as a scoring algorithm.

## Results and discussion

### UPLC-ESI-MS analysis for characterization of Cystoseira myrica, C. trinodis and C. tamariscifolia chloroform fractions

The metabolic profiling of the three species of *Cystoseira* is illustrated in (Table 1) and resulted in identification of 19, 20 and 11 compounds, respectively that belongs to different classes of secondary plant metabolites. The total ion chromatogram (TIC) in negative ionisation mode is represented in (Figure S2). The compounds were identified by analysing their mass data and comparing them with previously described compounds in existing literature.

**Table 1.** Metabolite profiling of *C. myrica*, *C. trinodis* and *C. tamariscifolia* Chloroform fraction using UPLC-ESI-MS in the negative ion mode.

Peak No.	Rt. (min)	Compound Name	[M − H] ^−^ (*m/z*)	Molecular Formula	Chemical class	Area %			References
						*C. myrica*	*C. trinodis*	*C. tamariscifolia*	
1	1.07	(+)-Afzelechin	273.00	C_15_H_14_O_5_	Flavonoid	1.37	1.60	1.73	^74^
2	1.36	Resveratrol	227.10	C_14_H_12_O_3_	Stilbene	10.04	22.18	–	^75^
3	1.91	(2E, l0E)-1-Hydroxy-6,13-diketo-7- methylene-3,11,15-trimethylhexadeca-2, l0, l4-triene	317.10	C_20_H_30_O_3_	Acyclic diterpene	0.76	2.03	–	^50^
4	2.19	6-Methoxy-4-methylcoumarin	189.15	C_11_H_10_O_3_	Coumarin	–	–	3.05	^76^
5	2.91	Syringic acid	197.85	C_9_H_10_O_5_	Phenolic acid	1.36	2.27	–	^75^
6	7.74	4′,7-dimethoxyluteolin	313.30	C₁₇H₁₄O₆	Flavonoid	–	–	2.63	77
7	8.64	Octadecanoic acid, ethyl ester	311.30	C_20_H_40_O_2_	Fatty acid	–	–	4.58	^78^
8	9.42	Cystomexicone B	357.20	C_22_H_30_O_4_	Nor sesquiterpenoid	–	–	0.36	^72^
9	10.27	Sagerinic acid	719.45	C_36_H_32_O_16_	Lignan	5.66	3.19	–	^79^
10	11.15	Eicosaenoic acid	309.30	C_20_H37O_2_	Fatty acid	2.09	1.13	–	^80^
11	11.35	Naringin	579.40	C_27_H_32_O_14_	Flavonoid	–	0.80	–	^81^
12	11.65	Compound 5	251.10	C_16_H_28_O_2_	Meroditerpenoids	–	–	2.41	^52^
13	11.76	Neoeriotrin	595.40	C_27_H_32_O_15_	Flavonoid	–	0.18	–	^81^
14	12.14	4′,12-Di-*O*-Acetylcystone F	557.35	C_32_H_45_O_8_	Meroditerpenoids	3.58	1.06	–	^15^
15	12.15	*p*-Coumaric acid glucoside	325.30	C_15_H_17_O_8_	Phenolic acid	–	–	0.71	^77^
16	12.27	Docosanedioic acid	369.20	C_22_ H_42_ O_4_	Fatty acid	–	0.42	–	^82^
17	12.51	Catechin dimer-deoxyhexose	723.45	–	Catechin	1.67	3.20	–	^83^
18	12.99	Hydroxy-linolenic acid	293.25	C_18_H_30_O_3_	Fatty acid	3.47	0.59	–	^84^
19	13.22	1-*O*-Caffeoyl-3-*O*-sinapoylquinic acid	559.40	C_27_H_28_O_13_	Phenolic acid	1.79	3.72	–	^85^
20	13.44	Quercetin	301.25	C_15_H_10_O_7_	Flavonoid	3.30	–	–	^86^
21	13.54	Pachydictyol A	287.30	C_20_H_32_O	Diterpene	–	–	29.84	^12^
22	13.99	Phytol	295.30	C_18_H_32_O_3_	Diterpene	3.45	–	–	^51^
23	14.17	3[A]chlorobifuhalol hexacetate	535.40	C_24_H_21_ClO_12_	Phlorotannins	–	2.47	–	^57^
24	14.45	(2E,10E)-1,6-dihydroxy-7-methy1ene-13-keto-3,11, 15-trimethylhexadeca-2,10,14-triene	319.30	C_20_H_32_O_3_	Acyclic diterpene	3.83	2.10	–	^50^
25	14.62	Quercetin 7-O-galloyl-hexoside	585.40	C_28_H_24_O_16_	Flavonoid	1.00	0.81	–	^87^
26	15.32	Dihydroxy hexadecanoic acid	287.30	C_16_H_31_O_4_	Fatty acid	3.79	–	2.86	^80^
27	15.45	2′′,3′′-Dihydro-3′,3′′′-biapigenin methyl ether	553.35	C_31_H_22_O_10_	Flavonoid	2.52	7.49	–	^88^
28	15.67	Alternariol 9-methyl ether	271.25	C_15_H_12_O_5_	Dibenzo-α-pyrone analog	8.87	–	–	^89^
29	15.89	Salvianolic acid F isomer	313.30	C_17_H_14_O_6_	Stilbene	–	0.98	1.85	^79^
30	16.00	Isorhamnetin 7-O-[3-hydroxy-3 -methylglutaroyl]hexoside	621.50	–	Flavonoid	2.90	–	–	^90^
31	16.73	Cystophloroketal A	563.45	C_34_H_44_O_7_	Phloroglucinol − meroterpenoid hybrid	2.17	–	–	^54^
32	16.89	Lithospermic acid A	537.40	C_27_H_22_O_12_	Benzofuran	–	4.07	1.70	^79^
33	17.79	Cystophloroketal B	563.40	C_34_H_44_O_7_	Phloroglucinol − meroterpenoid hybrid	24.72	21.71	1.11	^54^
34	17.98	Octadecatrienoic acid	277.20	C_18_H_29_O_2_	Fatty acid	–	–	2.03	^80^

Generally, the tentatively identified compounds belong to terpenoids, flavonoids, phenolic acids and fatty acids. The terpenoids represent the major class of metabolites in the chloroform fraction of the three species. Two acyclic diterpenes were characterised as (2*E*, l0*E*)-1-hydroxy-6,13-diketo-7-methylene-3,11,15-trimethylhexadeca-2, l0, l4-triene **(3)** and (2*E*,10*E*)-1,6-dihydroxy-7-methy1ene-13-keto-3,11,15-trimethylhexadeca-2,10,14-triene **(24)** with molecular formula of C_20_H_30_O_3_ and C_20_H_32_O_3_ and mass ion peaks at 317.10 and 319.30, respectively which were characterised in *C. myrica* and *C. trinodis* and formerly identified from *C. crinita*^50^. Moreover, the mass ion peak at *m/z* 295.30, corresponding to the predicted molecular formula C_18_H_32_O_3_, was identified as phytol **(22)**, earlier characterised from *C. tamariscifolia*^51^.

Two meroditerpenoids were characterised with ion mass peaks at *m/z* 251.10 and 557.35 corresponding to the predicted molecular formula C_16_H_28_O_2_ **(12)** and C_32_H_45_O_8_ **(14)**, respectively, they were identified previously from *C. baccata* and *C. usneoides*^15^^,^^52^ Whereas that at *m/z* 357.20 for the suggested molecular formula C_22_H_30_O_4_ was dereplicated as nor sesquiterpenoid; cystomexicone A or B **(8)** that was formerly characterised from *C. abies marina*^53^. Diterpene pachydictyol A **(21)** was dereplicated as major component in *C. tamariscifolia* with ion mass peak at *m/z* 287.30 corresponding to the molecular formula C_20_H_32_O. This compound was formerly isolated from *C. myrica*^12^. Phloroglucinol − meroterpenoid hybrids were characterised as cystophloroketal A and B **(31 & 33)** with same mass ion peak at *m/z* 563.45 corresponding to the predicted molecular formula C_34_H_44_O_7_, were isolated previously from *C. tamariscifolia*^54^. Regarding polyphenolic compounds as phenolic acids and flavonoids, the mass ion peaks at *m/z* 197.85, 325.30 and 559.40 in accordance with the molecular formula C_9_H_10_O_5_, C_15_H_17_O_8_ and C_27_H_28_O_13_, were dereplicated as syringic acid **(5)**, *p*-coumaric acid glucoside **(15)** and 1-O-caffeoyl-3-O-sinapoylquinic acid **(19)**, respectively where phenolic acids were previously characterised from brown algae different species as *Thalassiophyllum*, *Fucus* and *Cystoseira*^55^^,^^56^. Moreover, the identified flavonoids were more abundant in *C. myrica* and *C. trinodis* characterised as quercetin and its gallic acid derivative (**20 & 25**), naringin **(11)**, neoeriotrin **(13)** while 4′,7-dimethoxyluteolin **(6)** was dereplicated in *C. tamariscifolia* as shown in (Table 1). Another ion mass peak at *m/z* 535.40 was dereplicated as 3[A]chlorobifuhalol hexacetate **(23)** which belongs to phlorotannins and in agreement with the molecular formula of C_24_H_21_ClO_12_ that was previously identified from the brown algae *Carpophyllum angustifolium* (Sargassaceae)^57^.

**Figure 1.**
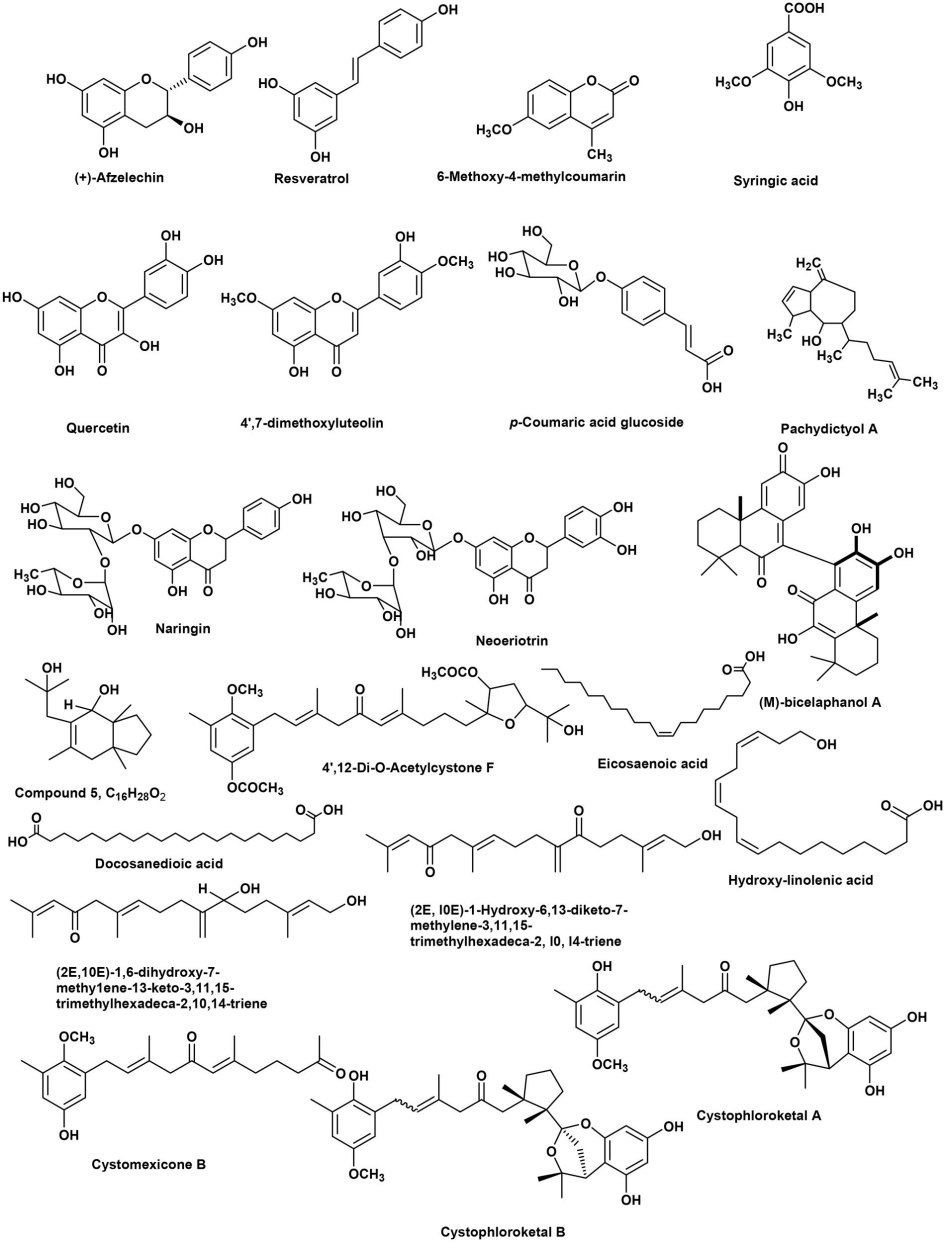
Chemical structures of the major characterised metabolites in three *Cystoseira* species using UPLC-ESI-MS in the negative ion mode.

Furthermore, six fatty acids were characterised in the three species namely, eicosaenoic acid **(10)**, docosanedioic acid **(16)** and hydroxy-linolenic acid **(18)**. Where, octadecanoic acid, ethyl ester **(7)**, dihydroxy hexadecanoic acid **(26)** and octadecatrienoic acid **(33)** identified specifically in *C. tamariscifolia.* Fatty acids especially the polyunsaturated fatty acids representes as main metabolites as formerly reported in six different *Cystoseira* spp.^58^. The chemical structures of the major characterised metabolites in three *Cystoseira* spp. are illustrated in (Figure 1).

### Total phenolics and flavonoids content

The total phenolic and flavonoid content of the chloroform fraction of *C. myrica*, *C. trinodis* and *C. tamariscifolia* were calculated as mg/g gallic acid equivalents (GAE) and mg/g quercetin equivalents (QE), respectively and the results are represented in **(**Table 2). The chloroform fraction of *C. trinodis* displayed the highest total phenolic content (TPC) 17.51 ± 0.85 mg/g GAE, followed by *C. myrica* (16.75 ± 1.03 mg/g GAE) and *C. tamariscifolia* (13.34 ± 0.62 mg/g GAE). The total flavonoid content values exhibited comparable patterns to the total phenolic content values. Where, the chloroform fraction of *C. trinodis* exhibited the highest total flavonoid content (TFC) 35.48 ± 3.14 mg/g QE, followed by *C. myrica* (25.41 ± 0.84 mg/g QE) and the lowest TFC was that of *C. tamariscifolia* (9.76 ± 1.25 mg/g QE). A previous study by Mansur et al. 2020 on *C. tamariscifolia* collected from shallow subtidal at Hannafore Point, Cornwall, UK revealed that the chloroform fraction TFC ranged from 16.69 ± 0.52 to 49.21 ± 4.83 in the four seasons and TPC ranged from 7.81 ± 0.31 to 68.75 ± 2.79 in the four seasons, where the highest content in the Spring^16^.

**Table 2.** Total phenolic and flavonoid content of *C. myrica*, *C. trinodis* and *C. tamariscifolia* chloroform fraction.

Extracts	Total phenolics (mg/g gallic acid equivalents)	Total flavonoids (mg/g quercetin equivalents)
*C. myrica*	16.75 ± 1.03	25.41 ± 0.84
*C. trinodis*	17.51 ± 0.85	35.48 ± 3.14
*C. tamariscifolia*	13.34 ± 0.62	9.76 ± 1.25

Values are mean ± SEM, *n* = 3

### In vitro biological activities

#### Antioxidant activity using DPPH and FRAP assay

Plants are believed to hold potential as a valuable resource for uncovering new and unique compounds. By exploring natural plants and isolating their compounds, we can come across numerous biologically active substances that serve as promising starting points for developing medications. That would be achieved through investigation of the secondary metabolites and plant extracts potential in the various cytotoxic, antioxidant, anti-inflammatory, antimicrobial and antiviral assays^4^^,^^59–61^. The antioxidant potential of the three chloroform fractions under study revealed a potent efficacy on DPPH and FRAP radical scavenging as illustrated in **(**Table 3). The IC_50_ value of the chloroform fraction of *C. trinodis* (13.79 ± 1.09 *µ*g/mL) for DPPH assay was comparable to that of the standard drug ascorbic acid (10.22 ± 0.64 *µ*g/mL). Also, *C. trinodis* showed significantly stronger FRAP scavenging potency (IC_50_ = 26.32 ± 1.97 *µ*g/mL) than the *C. tamariscifolia*. On the other hand, *C. myrica* exhibited the lowest antioxidant potential in DPPH assay and no activity on FRAP assay. Resveratrol is widely known as an antioxidant, anti-inflammatory molecule^62^ that is present in high percentage in *C. trinodis*. Resveratrol showed the most powerful inhibition with an EC_50_ value of 85 ± 2.7 µM in DPPH scavenging radical assay^63^. Also, syringic acid and 1-*O*-caffeoyl-3-*O*-sinapoylquinic acid are phenolic acids well known for their antioxidant properties^64^. In addition to the contribution of phenolics to the anti-oxidant properties of *C. trinodis*, the presence of mertoterpenoids, specifically cystophloroketal B, enhances its antioxidant activity^65^.

**Table 3.** Antioxidant activity (IC_50_) of *C. myrica*, *C. trinodis* and *C. tamariscifolia* chloroform fraction using DPPH and FRAP assays.

Extracts	IC_50_ (*µ*g/mL)	
	Scavenging ability on DPPH radicles	Scavenging ability in FRAP assay
*C. myrica*	494.19 ± 14.23	NA
*C. trinodis*	13.79 ± 1.09	26.32 ± 1.97
*C. tamariscifolia*	150.07 ± 8.12	284.73 ± 15.21
Ascorbic acid (Standard)	10.22 ± 0.64	20.89 ± 1.25

Values are mean ± SEM, *n* = 3

IC_50_: Inhibitory concentration 50%

NA: No activity

Previous studies investigated the antioxidant activity of different *Cystoseira* spp.^5^^,^^6^^,^^66^^,^^67^. Abu-Khudir et al. 2021 found that the 80% methanol extract of *C. crinita* exhibited antioxidant activity, as demonstrated by its performance in DPPH and ABTS assays. The extract displayed IC_50_ values of 125.6 *µ*g/mL and 254.8 *µ*g/mL for DPPH and ABTS, respectively^6^. Čagalj et al. 2022 reported the antioxidant activity of *C. compressa* across different seasons using different assays and the results revealed that all samples exhibited strong free radical scavenging activity and DPPH inhibition, with values exceeding 80%. Notably, the August sample demonstrated the highest activity, as evidenced by its ORAC value of 72.1 ± 1.2 *µ*M TE^66^. Also, another study by Mhadhebia et al. reported the antioxidant activity of three *Cystoseira* spp. namely, *C. crinita, C. sedoides* and *C. compressa* by using DPPH and FRAP assays^7^. In DPPH assay, the aqueous extract of *C. compressa* showed highest potency followed by *C. crinita* and *C. sedoides* with IC_50_ values of 12.0 ± 0.7, 20.0 ± 0.5 and 75.0 ± 0.8 *µ*g/mL, respectively as compared to Trolox with IC_50_ of 90.0 ± 0.2 *µ*g/mL. In the FRAP assay, *C. compressa* exhibits a significantly higher capacity to eradicate Fe^3+^ compared to *C. crinita*, with values of 2.6 and 0.9 mg GAE/g dried sample, respectively.

#### Anti-hyperglycaemic activity through α-amylase and α-glucosidase inhibition

The efficacy of the three tested chloroform fractions towards the inhibition of *α*-glucosidase and *α*-amylase are recognised as one of the most important enzymes involved in diabetes management. Many reports revealed the dependence on natural products to control the hyperglycaemia associated with type 2 diabetes mellitus especially through inhibition of *α*-glucosidase and *α*-amylase enzymes^40^^,^^68–70^. As shown in (Table 4**)**, In terms of our findings, the chloroform fraction of *C. trinodis* showed remarkable inhibition of *α*-glucosidase and *α*-amylase with an IC_50_ value of 20.29 ± 1.87 and 24.81 ± 2.34 *µ*g/mL, respectively. Followed by *C. tamariscifolia* with an IC_50_ value of 28.51 ± 1.87 and 46.29 ± 2.91 *µ*g/mL, respectively. While the lowest activity was recognised for *C. myrica* with an IC_50_ value of 46.91 ± 3.68 and 68.71 ± 4.09 *µ*g/mL. the results were compared to the standard drug acarbose (IC_50_ = 3.12 ± 0.28 and 12.29 ± 0.73 *µ*g/mL) towards *α*-glucosidase and *α*-amylase, respectively (Figure S3). A previous study revealed that resveratrol, a key component of *C. trinodis*, had an IC_50_ values of 376.22 and 99.18 nm against α-amylase and α-glucosidase, respectively^65^. These values indicate the highest activity of *C. trinodis* compared to other species examined in the study. Previous study by Çelenk and Sukatar (2020) showed that three Cystoseira spp. namely, *C. barbata, C. compressa* and *C. crinita* have a potent *α*-glucosidase inhibition rates 90.7 ± 0.01, 89.8 ± 0.02 and 91.9 ± 0.00, respectively^8^.

**Table 4.** Anti-hyperglycaemic activity and Anti-inflammatory activity % (IC_50_) of *C. myrica*, *C. trinodis* and *C. tamariscifolia* chloroform fraction.

Extracts	IC_50_ (*µ*g/mL)		
	α-Amylase	α-Glucosidase	COX-1
*C. myrica*	68.71 ± 4.09	46.91 ± 3.68	19.76 ± 1.46
*C. trinodis*	24.81 ± 2.34	20.29 ± 1.87	16.13 ± 0.89
*C. tamariscifolia*	46.29 ± 2.91 µg	28.51 ± 1.87	52.19 ± 3.12
Acarbose (Standard)	12.29 ± 0.73	3.12 ± 0.28	–
Celecoxib (Standard)	–	–	9.07 ± 0.67

Values are mean ± SEM, *n* = 3

IC_50_: Inhibitory concentration 50%

NA: No activity

#### Anti-inflammatory activity

The anti-inflammatory potential of the three chloroform factions was investigated as the inflammation process is linked to many chronic ailments as ageing, gastritis, diabetes, hypertension, cardiovascular disease, rheumatoid and cancer^71^^,^^72^. As presented in (Table 4), the findings were evaluated using the percentage of inhibition and given as mean ± standard deviation and compared to the standard drug clelcoxibe with an IC_50_ value equals to 9.07 ± 0.67 *µ*g/mL. The results revealed that the three chloroform fractions showed a promising anti-inflammatory activity through inhibition of COX-1 expression. It was notable that the chloroform fraction of *C. trinodis* showed the highest activity with IC_50_ =16.13 ± 0.89, followed by *C. myrica* IC_50_ =19.76 ± 1.46 and *C. tamariscifolia* had the lowest anti-inflammatory efficacy IC_50_ =52.19 ± 3.12 *µ*g/mL (Figure S4). Our results were in accordance with the previous reports concerning the anti-inflammatory effects of different *Cystoseira* spp. ^7,^^8^^,^^71^. The extracts obtained from *Cystoseira amentacea* var. *stricta* using ethanol and DMSO exhibited potent inhibition of NO overproduction, with EC_50_ values ranging from 546 to 1293 µg/mL. Moreover, these extracts strongly suppressed the production of inflammatory mediators such as IL-1, IL-6, cyclooxygenase-2 and inducible NO synthase gene expression in RAW 264.7 macrophages that were induced by LPS^71^. Another study by Mhadhebia et al. reported the anti-inflammatory potency of three *Cystoseira* spp. namely, *C. crinita, C. sedoides* and *C. compressa.* The three species revealed anti-inflammatory activity *in-vivo*, using carrageenan induced rat paw oedema assay at doses of 25 or 50 mg/Kg i.p.^7^. The findings of the current study are consistent with earlier reports on various species of *Cystoseira* that have demonstrated antioxidant, antidiabetic, and anti-inflammatory effects.

### Molecular modelling

This part was carried out to explore the potential mechanism by which the identified key compounds exert their biological effects. Accordingly, the 3D structures of *α*-amylase, *α*-glucosidase, COX-1 & tyrosinase enzymes were obtained from the Protein Data Bank (PDB) with the following IDs: 7TAA, 7KB8, 5WBE and 5M8Q, respectively. Subsequently, the sixteen major compounds were subjected to docking within the vicinity of the active sites of the four enzymes. Remarkably, all the compounds exhibited satisfactory binding scores during the docking process with the four proteins (Table 5).

**Table 5.** The docking scores achieved by the major identified compounds towards various enzymes.

Compound Name	Docking Score (Kcal/Mol)			
	*α*-Amylase (7TAA)	*α*-Glucosidase (7KB8)	COX-1 (5WBE)	Tyrosinase (5M8Q)
Resveratrol	−12.51	−11.42	−12.86	−9.59
(2E, l0E)-1-Hydroxy-6,13-diketo-7- methylene-3,11,15-trimethylhexadeca-2, l0, l4-triene	−10.17	−12.75	−12.69	−7-07
6-Methoxy-4-methylcoumarin	−8.23	−8.66	−10.19	−7.62
4′,7-dimethoxyluteolin	−13.69	−13.38	−17.05	−9.83
Eicosanoic acid	−10.11	−10.69	−12.17	−9.15
Naringin	−13.91	−17.24	−20.29	−11.51
Hydroxy-linolenic acid	−10.03	−11.69	−12.97	−9.90
Quercetin	−13.52	−13.32	−15.43	−13.01
Pachydictyol A	−10.22	−12.81	−12.41	−8.52
Phytol	−9.28	−10.00	−12.01	−10.56
(2E,10E)-1,6-dihydroxy-7-methy1ene-13-keto-3,11,15-trimethylhexadeca-2,10,14-triene	−9.40	−10.53	−12.71	−7.81
2′′,3′′-Dihydro-3′,3′′′-biapigenin methyl ether	−14.93	−14.88	−18.07	−14.81
Compound 5	−8.42	−10.71	−10.92	−7.83
4′,12-Di-*O*-Acetylcystone F	−12.56	−12.09	−16.11	−9.51
Cystophloroketal A	−10.87	−12.91	−16.09	−11.23
Cystophloroketal B	−12.22	−12.58	−7.72	−11.61

**Figure 2.**
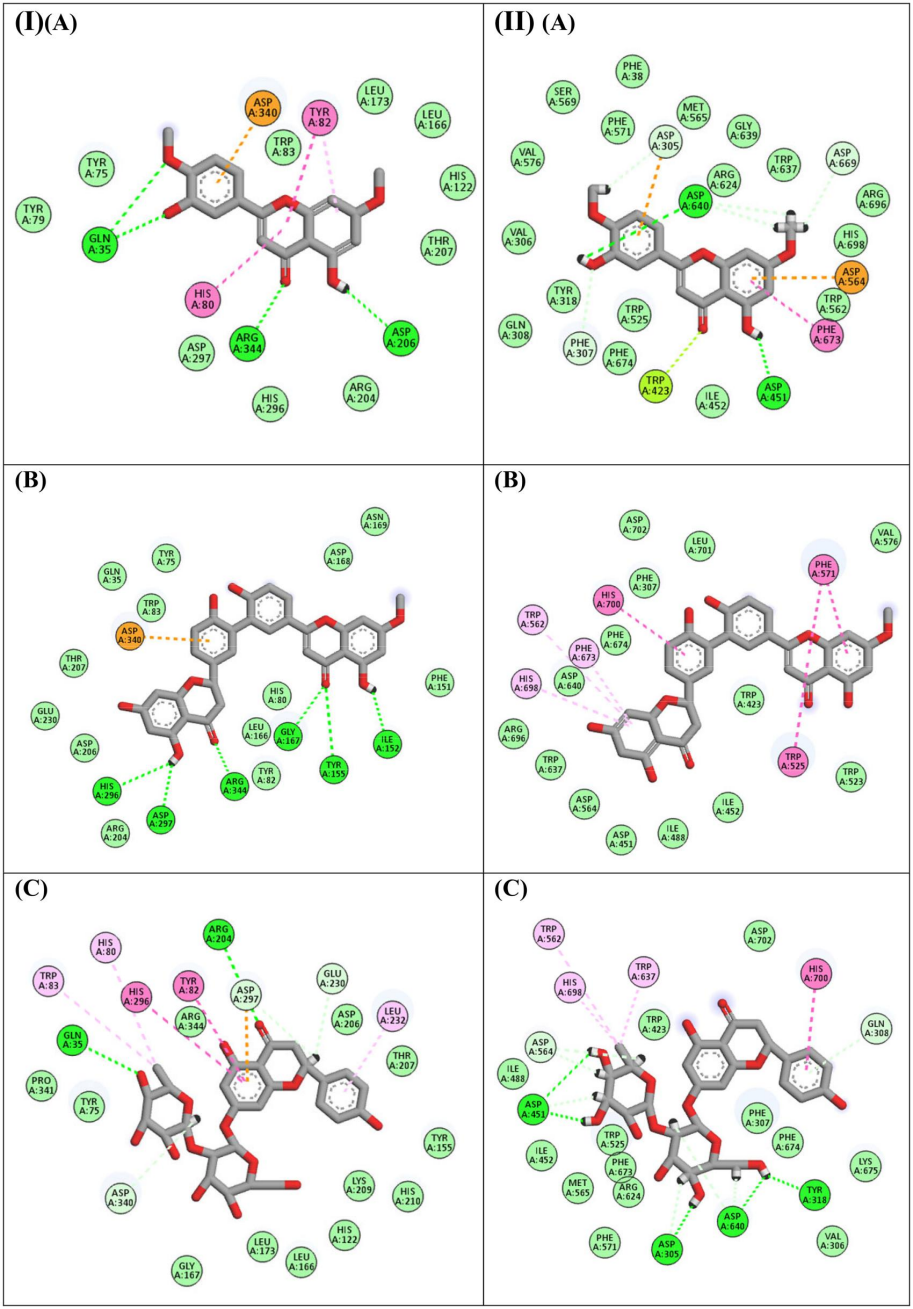
(I) 2D binding modes of (A) 4′,7-Dimethoxyluteolin, (B) 2″,3″-Dihydro-3',3'″-biapigenin methyl ether, (C) Naringin to the active binding sites of *α*-amylase enzyme. (II) 2D binding modes of (A) 4′,7-dimethoxyluteolin, (B) 2″,3″-dihydro-3',3'″-biapigenin methyl ether, (C) Naringin to the active binding sites of *α*-glucosidase enzyme.

Starting with the docking of the major compounds with the *α*-amylase, 2″,3″-dihydro-3′,3′″-biapigenin methyl ether, naringin, and 4′,7-dimethoxyluteolin were the best compounds achieving docking scores of −14.9, −13.9 and −13.6 Kcal/Mol, respectively. Inspecting the interaction with *α*-amylase (Figure 2), 2″,3″-dihydro-3′,3′″-biapigenin methyl ether formed six hydrogen bond interactions with Ile152, Tyr155, Gly167, His296, Asp297, and Arg344, in addition to a hydrophobic interaction with Asp340. Naringin formed two hydrogen bond interactions with Gln35 and Arg204, in addition to group of hydrophobic interactions with His80, Tyr82, Trp83, Glu230, Leu232, His296, Asp297 and Asp340, while 4′,7-dimethoxyluteolin formed four hydrogen bond interactions with Gln35, Asp206 and Arg344, in addition to hydrophobic interactions with Gly584, Asp586 and Trp801.

Regarding the docking of the major compounds with the *α*-glucosidase, naringin, 2′',3″-Dihydro-3′,3′″-biapigenin methyl ether, and 4′,7-dimethoxyluteolin were the best compounds achieving docking scores of −17.24, −14.88 and −13.38 Kcal/Mol, respectively. Inspecting (Figure 2), it was found that, naringin formed five hydrogen bond interactions with Asp304, Tyr318, Asp451 and Asp640, besides it formed several hydrophobic interactions with Gln308, Trp562, Asp564, Trp637, His698 and His700, 2″,3″-Dihydro-3′,3′″-biapigenin methyl ether formed many hydrophobic interaction with Trp525, Trp562, Phe571, Phe673, His698 and His700, while 4′,7-dimethoxyluteolin formed two hydrogen bond interactions with Asp451 and Asp640, moreover it formed several hydrophobic interactions with Asp305, Phe307, Trp423, Asp564, Asp669 and Phe673.

Sahnoun et al. reported that the α-amylase’s binding site for naringin exhibited a higher polar contact count, including 47 more than the count observed with acarbose. The binding site of naringin with α-glucosidase exhibited a higher number of polar contacts (45 interactions) compared to acarbose (39 interactions). Acarbose has less residues compared to other substances. The docking investigations indicate that naringin has a greater inhibitory action against α-glucosidase compared to acarbose. The naringin-α-glucosidase enzyme maintains a relatively constant amount of Rg, resulting in the formation of a more stable structure^73^.

**Figure 3.**
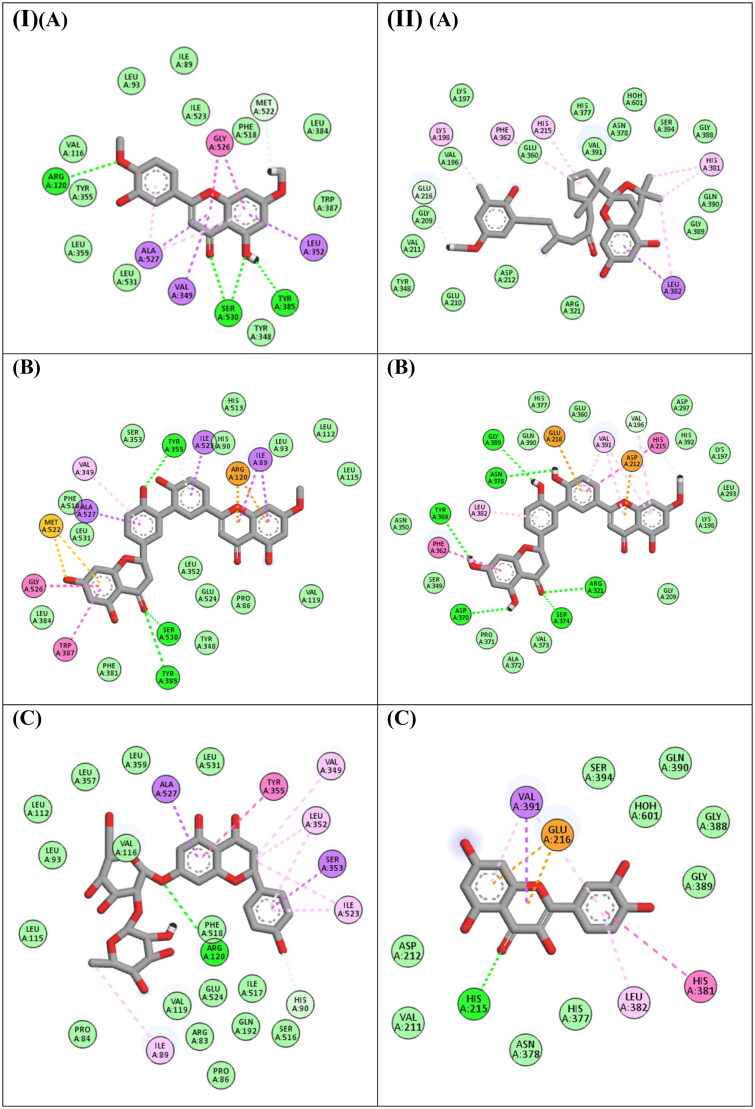
(I) 2D binding modes of (A) 4',7-Dimethoxyluteolin, (B) 2'',3''-Dihydro-3',3'''-biapigenin methyl ether, (C) Naringin to the active binding sites of COX-1 enzyme. (II) 2D binding modes of (A) Cystophloroketal B, (B) 2'',3''-Dihydro-3',3'''-biapigenin methyl ether, (C) Quercetin to the active binding sites of Tyrosinase enzyme.

Moreover, the docking investigation of the major compounds with COX-1, naringin, 2″,3″-dihydro-3′,3′″-biapigenin methyl ether, and 4′,7-dimethoxyluteolin were the best compounds achieving docking scores of −20.29, −18.07 and −17.05 Kcal/Mol, respectively. Inspecting (Figure 3), it was found that, naringin formed one hydrogen bond interaction with Arg120, and it formed several hydrophobic interactions with Ile89, His90, Val349, Leu352, Ser353, Tyr355, Ile523, and Ala527, 2″,3″-Dihydro-3′,3′″-biapigenin methyl ether formed three hydrogen bond interactions with Tyr355, Tyr385 and Ser530, additionally, it formed several hydrophobic interactions with Ile89, Arg120, Val349, Trp387, Met522, Ile523, Gly526 and Ala527, while 4′,7-dimethoxyluteolin formed four hydrogen bond interactions with Arg120, Tyr385 and Ser530, moreover it formed several hydrophobic interactions with Val349, Leu352, Gly526 and Ala527.

Furthermore, in the docking of the major compounds with tyrosinase, 2′',3′'-dihydro-3′,3′″-biapigenin methyl ether, quercetin and cystophloroketal B were the best compounds achieving docking scores of −14.81, −13.01 and −11.81 Kcal/Mol, respectively. Inspecting (Figure 3), it was found that, 2′',3″-dihydro-3′,3′″-biapigenin methyl ether was engaged in many hydrogen bond interaction with Arg321, Tyr369, Asp370, Ser374, Asn378 and Gly389, besides it formed several hydrophobic interactions with Val196, Asp212, His215, Glu216, Phe362, Leu382, and Val391. Quercetin formed one hydrogen bond interaction with His215 many hydrophobic interactions with Glu216, His381, Leu382, and Val391, while cystophloroketal B was able to engage with various hydrophobic interactions with Lys198, His215, Phe362, His381, and Leu382. According to a previous investigation, it was determined that quercetin had a maximum binding affinity of −7.9 kcal/mol towards tyrosinase. This interaction occurred within the hydrophobic pocket of the enzyme, which was encompassed by specific amino acid residues including Val-248, Met-257, Phe-264, Met-280, Val-283, and Ala-286. Consequently, a strong hydrophobic binding was established between quercetin and the residues. Furthermore, the presence of chelation processes was detected between the quercetin and the two copper atoms, representing the primary interaction between quercetin and tyrosinase^62^.

In conclusion, all the major compounds induced a favourable binding with the four targets, especially, 4′,7-dimethoxyluteolin, 2″,3″-dihydro-3′,3′″-biapigenin methyl ether, naringin, quercetin and cystophloroketal B.

## Conclusion

The results of this study indicate that the chloroform fraction of the three investigated *Cystoseira* spp. contain a various secondary metabolites belonging to terpenoids, flavonoids, phenolic acids, and fatty acids, that correlates with their antioxidant and inhibitory activities towards the tested enzymes. The chloroform fraction of *C. trinodis* showed the highest reducing property in DPPH and FRAP assays, also remarkable inhibition of *α-*glucosidase, *α*-amylase, and COX-1 expression. Moreover, through molecular docking studies, the identified major compounds offered a plausible mechanism prediction for the biological effects exerted by the chloroform fractions. Among the major compounds, 4′,7-dimethoxyluteolin, 2′',3″-dihydro-3′,3′″-biapigenin methyl ether, naringin, quercetin and cystophloroketal B induced a favourable binding score with the four enzymes. Eventually, the present study suggest that the *Cystoseira* spp. show promise as potential candidates for further exploration and the development of potential treatments for chronic conditions such as Alzheimer’s disease, diabetes, and oxidative stress-related disorders. These expected modes of action still need to be confirmed by further mechanistic investigation in the future.

## Supplementary Material

Supplemental Material
