# Supplementary material for: Comparative metabolic study of the chloroform fraction of three Cystoseira species based on UPLC/ESI/MS analysis and biological activities
Source: J Enzyme Inhib Med Chem. 2023 Dec 12;39(1):2292482. doi: 10.1080/14756366.2023.2292482 (PMC11721769; doi:10.1080/14756366.2023.2292482)
Supplement: Supplemental Material [file IENZ_A_2292482_SM6113.pdf]

Supplementary material

Comparative Metabolic Study of The Chloroform Fraction of Three *Cystoseira* species Based on UPLC/ESI/MS Analysis and Biological Activities

Shaza H. Aly[1,*], Ahmed M. Elissawy[2, 3], Mahmoud A. El Hassab[4], Taghreed A. Majrashi[5], Fatma E. Hassan[6,7], Eslam B. Elkaeed[8], Wagdy M. Eldehna[9,*], Abdel Nasser B. Singab [2,3,*]

[1] *Department of Pharmacognosy, Faculty of Pharmacy, Badr University in Cairo (BUC), Badr City, Cairo 11829, Egypt*
[2] *Department of Pharmacognosy, Faculty of Pharmacy, Ain-Shams University, Cairo 11566, Egypt*
[3] *Centre of Drug Discovery Research and Development, Ain Shams University, Cairo 11566, Egypt*
[4] *Department of Medicinal Chemistry, Faculty of Pharmacy, King Salman International University (KSIU), South Sinai 46612, Egypt*
[5] *Department of Pharmacognosy, College of Pharmacy, King Khalid University, Asir 61421, Saudi Arabia.*
[6] *Department of Physiology, General Medicine Practice Program, Batterjee Medical College, Jeddah 21442, Saudi Arabia*
[7] *Medical Physiology Department, Kasr Alainy, Faculty of Medicine, Cairo University, Giza 11562, Egypt*
[8] *Department of Pharmaceutical Sciences, College of Pharmacy, AlMaarefa University, Riyadh 13713, Saudi Arabia*
[9] *Department of Pharmaceutical Chemistry, Faculty of Pharmacy, Kafrelsheikh University, Kafrelsheikh 33516, Egypt*

[*]Corresponding author: S.H.A. shaza.husseiny@buc.edu.eg; W.M.E. wagdy2000@gmail.com; A.N.B. S. dean@pharma.asu.edu.eg

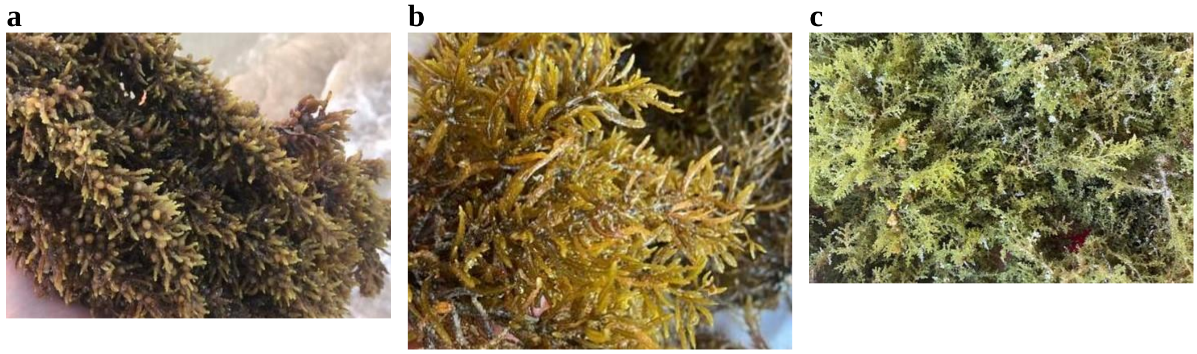

Figure S1. Images of the three samples under study; **a.** *Cystoseira myrica*, **b.** *Cystoseira trinodis*, **c.***Cystoseira tamariscifolia*.

(a)

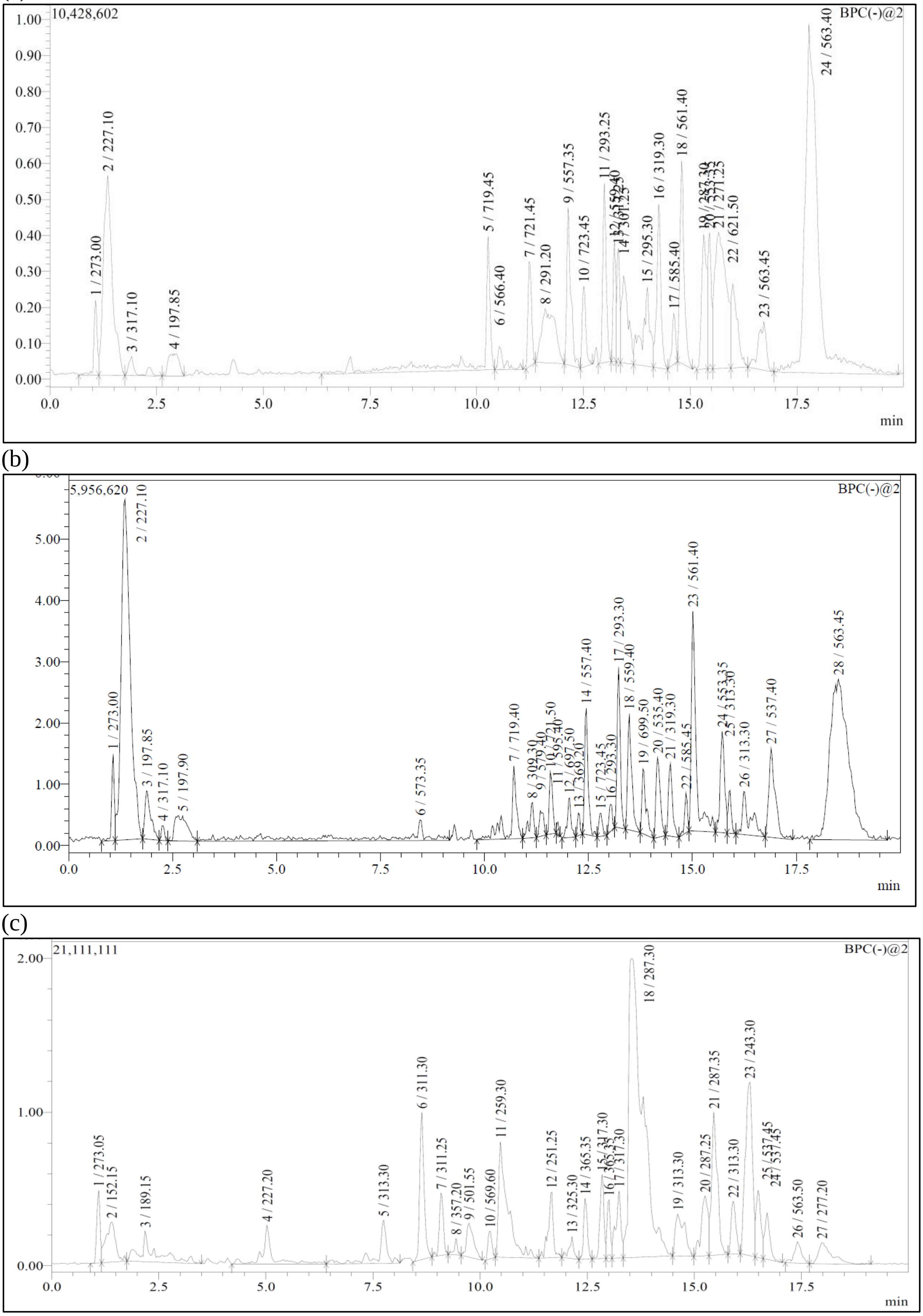

Figure S2. Total ion chromatogram (TIC) for *C. myrica* (a), *C. trinodis* (b) and *C. tamariscifolia* (c) chloroform fraction using UPLC/ESI/MS in the negative ion mode.

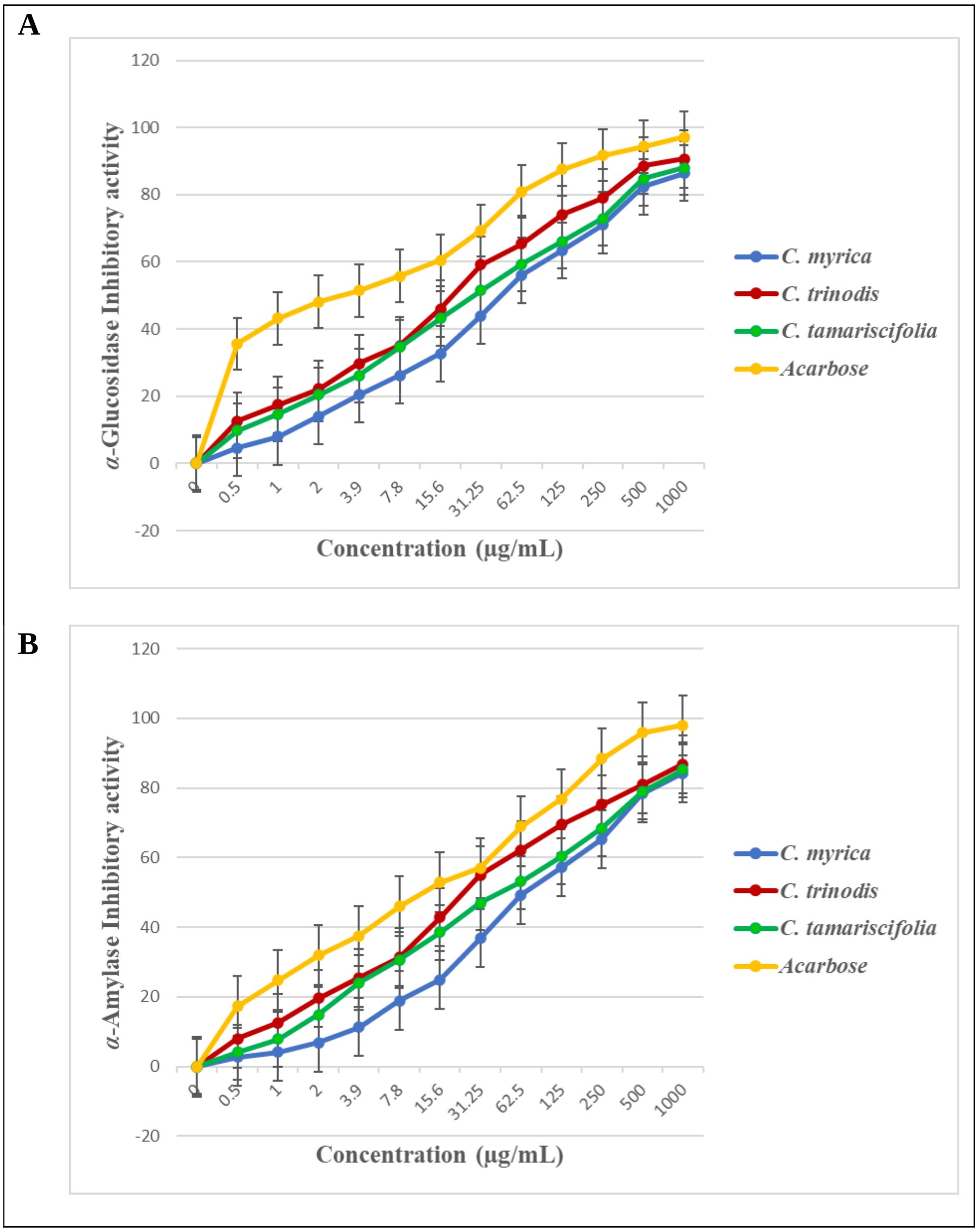

Figure S3. Anti-hyperglycaemic activity: **A)** α-glucosidase and **B)** α-amylase inhibitory % of *C. myrica*, *C. trinodis* and *C. tamariscifolia* compared to standard drug, Acarbose.
Values are means of three determinations, mean ± SEM

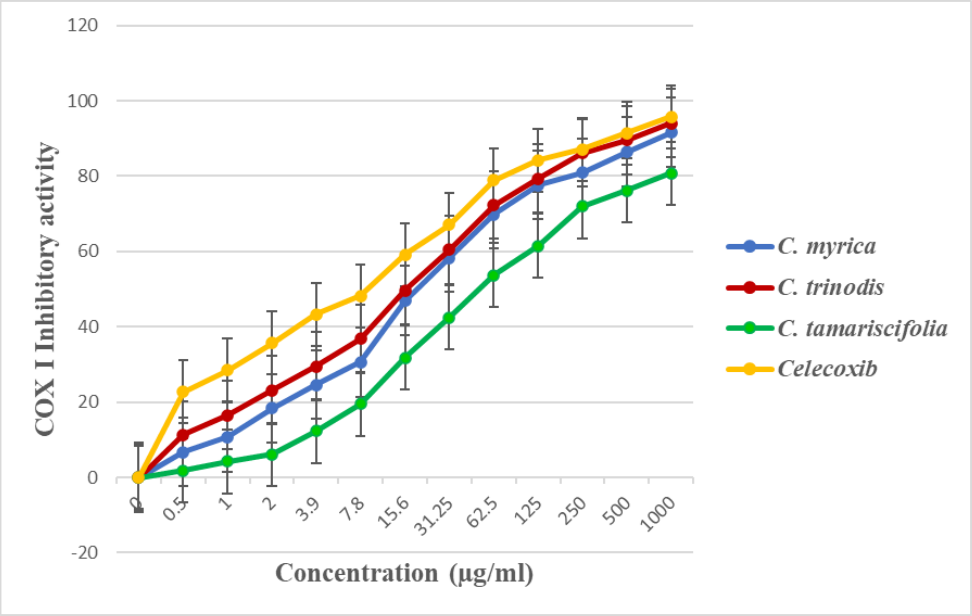

Figure S4. Anti-inflammatory activity cyclooxygenase COX-1 inhibitory % of *C. myrica*, *C. trinodis* and *C. tamariscifolia* compared to standard drug, celecoxib.

Values are means of three determinations, mean ± SEM; COX-1: Cyclooxygenase-1
